# Neuronal Cell Reconstruction with Umbilical Cord Blood Cells in the Brain Hypoxia-Ischemia

**DOI:** 10.6091/ibj.1376.2015

**Published:** 2015-01

**Authors:** Hossein Ali Ghaffaripour, Mehdi Jalali, Mohammad Reza Nikravesh, Masoumeh Seghatoleslam, Javad Sanchooli

**Affiliations:** 1 *Dept. of Pediatrics, Medical School, Zabol Medical Science University, Zabol, Iran. *; 2 *Dept. of Anatomy and Cell Biology, Medical School, Mashhad University of Medical Sciences, Mashhad, Iran. *; 3 *Dept. of Biochemistry and Immunology, Medical School, Zabol Medical Science University, Zabol, Iran*

**Keywords:** Hypoxia-Ischemia, Nerve cell, Umbilical Cord Blood

## Abstract

**Background:** Brain hypoxia-ischemia is a human neonatal injury that is considered a candidate for stem cell therapy. **Methods:** The possible therapeutic potential of human umbilical cord blood (HUCB) stem cells was evaluated in 14-day-old rats subjected to the right common carotid occlusion, a model of neonatal brain hypoxia-ischemia. Seven days after hypoxia-ischemia, rats received either saline solution or 4 × 10^5^ HUCB cells i.v. Rats in control group did not receive any injection. After two weeks, rats were assessed using two motor tests. Subsequently, rats were scarified for histological and immunohistochemical analyses. **Results:** Our immunohistochemical findings demonstrated selective migration of the injected HUCB cells to the ischemic area as well as reduction in infarct volume. Seven days after surgery, we found significant recovery in the behavioral performance in the test group (12.7 +/- 0.3) compared to the sham group (10.0 +/-0.05), a trend which continued to day 14 (15.3 ± 0.3 vs. 11.9 ± 0.5, *P*<0.05). Postural and motor asymmetries at days 7 and 14 in the test group showed a significant decrease in the percentage of right turns in comparison to the sham group (75% and 59% vs. 97% and 96%, *P*<0.05). **Conclusion: **The results show the potential of HUCB stem cells in reduction of neurologic deficits associated with neonatal hypoxia-ischemia.

## INTRODUCTION

Hypoxia-ischemia is major cause of fetal brain damage with long-lasting behavioral implic-ations that occurs in approximately 2-4 per 1,000 full-term births [1]. Among the surviving infants, up to 25% will have a permanent neurologic deficit in the new strategies of cerebral palsy, epilepsy, learning disability, or mental retardation [2]. In spite of hopeful neuroprotective strategies in animal models and also clinical trials, prevalent treatment methods are limited to supportive intensive care [3]. Furthermore, it is necessary to come up with new neuroprotective approaches that could reduce the neurologic deficit of hypoxia-ischemia in newborns. 

Over the past years, for cure of neurological disorders, cell transplantation has considered as a potential approach [4-6]. One of the proposed cells in this issue is human umbilical cord blood cells that are sources of hematopoietic stem/progenitor cells (HSC) [7]. In 1989, the first successful hematopoietic reconstitution with human umbilical cord blood (HUCB) stem cells has been reported in a child with Fanconi anemia, and then a large number of transplants have been performed for malignant and non-malignant hematological disorders [8]. Moreover, preclinical studies have shown that human umbilical cord mesenchymal cells injected systemically in the acute phase of animal models of stroke and the hypoxia-ischemia-insulted animals can migrate toward ischemic regions and cross the blood brain barrier [9] and have therapeutic effect. These cells can reduce the area of brain infarction [10] and increase the regenerative capacity of the brain [11], thus improving behavioral recovery [12-15]. On the other hand, mononuclear cells acquired from HUCB containing HSC are easy to obtain, ethically unproblematic and available for allogeneic approaches. Therefore, these HSC can be suitable candidates for cell therapies [16]. 

Although several groups have reported beneficial effects of human umbilical cord blood cells administration after neonatal hypoxia-ischemia [17, 18], there has been a shortage of studies on the therapeutic benefit of these cells on hypoxia-ischemia. In the current study, the potential of HUCB in reducing the neurological deficits associated with neonatal hypoxia-ischemia was examined by evaluation of their impact on motor performance and brain morphologic changes. 

## MATERIALS AND METHODS

***The isolation of mononuclear cells and BrdU labeling.*** After the approval of study by the Ethical Committee of Mashhad University of Medical Sciences (Mashhad, Iran), mothers' umbilical cord blood with a range of 20-40 years was collected in special bags containing dextrose adenine citrate phosphate. Women who had no history of smoking, alcohol, or especial disease were selected from the Obstetrics and Gynecology Department of Ghaem Hospital, Mashhad, Iran. Blood samples were rapidly diluted 1:1 in PBS and diluted again to a ratio of 8:3 in 15-ml centrifuge tubes with Ficoll-Paque centrifug-ation at 800 ×g at room temperature (RT) for 20 min. Next, cloudy mononuclear cell interface layer (Buffy coat) was carefully removed by pipetting, transferred to a new tube, washed twice with PBS through centrifugation at 800× g for 10 to 20 min in RT and then the cells were resuspended in 1 ml UCB serum. Mononuclear cells were then nurtured with culture medium RPMI supplemented with 10% fetal bovine serum and 10 ml/L antibiotic. Next, the cells were labeled with 3 μg/ml BrdU and incubated at 37°C for 24 hours. Finally, they were counted using a Neubauer hemocytometer plate, and their viability was estimated using the Trypan blue dye exclusion method. 

***Brain ischemia*** ***modeling****. *A number of 20 Wistar rats (two weeks old) were selected in this study. All animals were housed in a room under a constant temperature (22 ± 2ºC) and illuminated 7:00 A.M. to 7:00 P.M. with food pellets and water available *ad libitum*. Animal handling and all related procedures were carried out in accordance with Mashhad University of Medical Sciences, Ethical Committee Acts (Mashhad, Iran). Hypoxic ischemia model was created based on Hidetoshi's method. Briefly, rats were anesthetized with 30 mg/kg ketamine and 4 mg/kg xylazine (i.p.), and the right common carotid artery was permanently ligated. Thereafter, the pups were placed in a chamber maintained at 37°C, through which humidified 8% oxygen and balanced nitrogen were flowed for 1-2 h [19]. Then, the skin incision was sutured, and animals were kept under sterile conditions until recovery. 

***Experimental protocol*** *. *Ten neonates were received i.v. 2 × 10^5^ stem cells at seven days after the hypoxia-ischemia as test group. The sham group was considered 10 neonates with hypoxic conditions that did not receive any stem cells, and 10 healthy pups without hypoxia were placed in control group.

***Investigation of motor and behavioral change*** *. *Two behavioral tests were performed for all 30 rats three times on days 1, 7, and 14 after stem cell injection. These tests, including limb placing test [20] and corner turn test [21] were evaluated by an observer blinded to. group designation. The limb placing test consisted of 4 tasks that evaluated the sensory motor integration of the forelimb and the hind limb and checked tactile and proprioceptive stimulation feedback. Each test was scored as follows: 0, no placing; 1, incomplete and/or delayed (>2 seconds) placing; and 2, immediate and correct placing. The highest score of 16 was typically given to normal rats. During task 1, the rat was suspended 10 cm over a table, and the stretch of the forelimbs toward the table was observed and evaluated. Normal rats reached, stretched, and placed both forepaws on the table top. For task 2, visual and tactile contact with the table was avoided by supporting the rat’s chin and holding its head 45º upwards. In task 3, the rat was positioned along the edge of the table, with its forelimbs suspended over the edge and allowed to move freely. Each limb (forelimb and hindlimb) was gently pulled down, and retrieval and placement were checked. Task 4 recorded forelimb and hindlimb placement when the lateral side of the rat’s body was moved toward the table edge. In the corner turn test, the rats were led into a corner with an angle of 30º. For exiting the corner, the rats could turn either to the left or the right randomly. This leading was repeated and recorded 10 times with at least 30-s interval. Then, the percentage of right turns was calculated. Of course rearing movement of the rats was not part of turning and was not scored. 

***Histological and immunohistochemistry*** ***assess-ment****. *All rats were anesthetized (ketamine i.p.) 14 days after the last motor tests and perfused with 100 ml cold saline, followed by 100 ml paraformaldehyde 4% in 0.1 mol/l phosphatebuffered saline. Then, their brains were fixed in paraformal-dehyde fixative for 24 hours. The paraffin blocks were prepared from each brain and then were sectioned with six-micron thickness from blocks. Each 40^th^ section of the histological series was stained with hematoxylin and eosin. Volume of ischemic lesion in each section was calculated using image analysis system (Data Translation, Marlboro, and MA). The area of ischemia and the area of both hemispheres (mm^2^) were calculated by tracing the area on the computer screen, and the volumes (mm^3^) were determined by integrating the appropriate area with the section interval thickness. To reduce errors associated with processing of tissue for histological analysis, the area of infarction in each section was presented as the percentage of the ischemia to the area of the contralateral hemisphere. The ischemia volume was also presented as the percentage of ischemic volume to the volume of the contralateral hemisphere [22]. BrdU labeling was performed to trace transplanted stem cell in the stratum of damaged brain. The sections were incubated with anti-BrdU primary antibody and then with peroxidase-labeled secondary antibody. After that the sections were stained with diaminobenzidine solution and evaluated using a light microscope. Negative control sections from each animal were prepared for immunohistochemical staining. An average of 10 histology slides from third block of brain per test group was selected, and then an Olympus BX51 microscope equipped with camera and software Image J [23] was used for analyzing BrdU+ cells. All BrdU-reactive cells in the injured hemisphere were counted throughout all 10 coronal sections.

**Fig 1:**
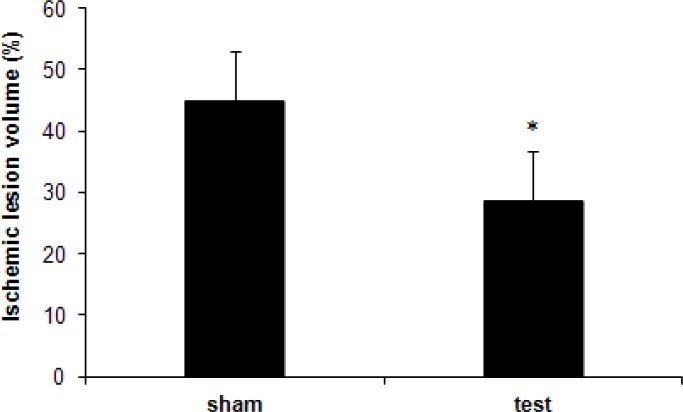
The percentage of ischemic volume to the volume of the contralateral hemisphere in the test and sham groups two weeks after injection. By day 14, there was a significant decrease in ischemic volume between the two groups (*P<0.05).

***Statistical analysis*** *. *The data were presented as mean ± SE. All behavioral and histological analyses were performed by experimenters blind to group identity. Histological data and scores of functional tests were analyzed using analysis of variance with Duncan's post hoc test. However, the analysis of variance was carried out for scores of functional tests on days 1, 7, and 14 separately. A *P*<0.05 was considered statistically signiﬁcant. 

## RESULTS

***Histological determination of ischemia volume percentage. ***The ischemic volume of brain on day 14 post-injection was smaller in hypoxia-ischemia rats treated with umbilical cord blood stem cells compared to that received vehicle. Significant differences in percentage of ischemic volume were detected between the test group (28.7 ± 6.7%) and the sham group (44.9 ± 7.4%) (*P*<0.05, Fig. 1). 

**Fig. 2:**
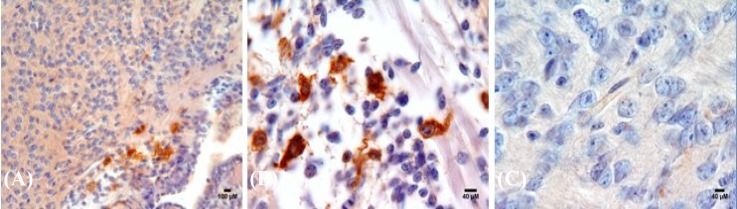
Histological images of infused human umbilical cord blood in the brain of test group (A and B). Coronal brain sections of 14-day-old rat in the group treated with labeled UCBSC appeared as a brown color compared with (C) negative control group.

***Qualitative analysis of human umbilical cord blood*** ***cell migration in the ischemic area.*** The presence of BrdU + cells in the test group was confirmed using immunohistochemistry. As shown in Figure 2 (A and B), the migrated labeled cells were appeared in the brown color in the damaged area. The number of migrated BrdU positive cells at 14^th^ day after injection in the test group was 560 ± 90.

**Fig. 3:**
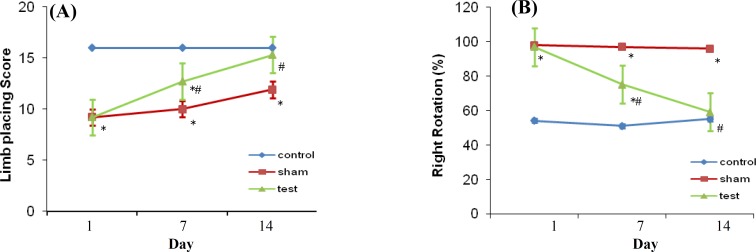
Behavioral tests performed on days 1, 7, and 14 after ischemia. (A) Animals in the test group exhibited significantly better performance in the limb-placing function (score ± SE) compared with the sham group at 7 and 14 days after UCBSC injection (*P<0.05 vs. control; # *P*<0.05 vs. sham). (B) Animals in test group exhibited a progressive decrease in ipsilateral (right) turns over the 14-day testing, and their results approached to the control animals. By day 14, there was a significant decrease in turning right between test and sham groups (*P<0.05 vs. control; # *P*<0.05 vs. sham).

***Behavioral recovery. ***There were no significant differences among the test and sham groups in limb placing (9.2 ± 0.2 vs. 9.2 ± 0.3, respectively) and corner turn test scores (97% vs. 98%, respectively) on day one after transplantation (Fig. 3). Rats treated with human UCBSC displayed significantly better performance on limb- placing test score compared with sham group on days 7 and 14 after transplantation (12.7 ± 0.3 vs. 10.0 ± 0.5, respectively and 15.3 ± 0.3 vs. 11.9 ± 0.5, respectively).

The scores of this test in control group were 16 points in each three times (1, 7, and 14 days). Concerning the scores of the test and sham group, the control group had significant difference with both groups at days first and 7^th^ after injection except at day 14; there was no significant difference between the control and test groups (Fig. 3A). In corner turn test, postural and motor asymmetries were tested by recording the numbers of turns to the right from 10 trials on days 1, 7, and 14 after ischemia, and the values were presented as percent. Compared to sham group, the percentage of the right turns on days 7 and 14 after transplantation was significantly decreased in rats transplanted with UCBSC (75% vs. 97% and 59% vs. 96%). On the other hands, the results of the control group on one and seven days after injection (51% and 55%) were significantly different from the two other groups. However, 14 days after ischemia, there was no significant difference between the control and test groups (Fig. 3b).

## DISCUSSION

Umbilical cord blood is a prominent source of non-embryonic multipotent stem cell that it has been shown these cells have the ability to regenerate numerous tissue types, and when transplanted into animals and humans, they produce measurable functional enhancement [24, 25]. Presently, in addition to its use for the treatment of hematological diseases, recent studies have focused on possible therapeutic effect of these cells in different models of central nervous system damage, including stroke in adult animals [9, 12, 14]. Several groups have reported that HUCB cells delivered obviously enhance functional recovery after ischemic injury in adult rats. The mechanisms of this recovery are not known but may refer to cytokines and trophic factors produced by HUCB cells [10, 12, 26]. 

A cascade of inflammatory molecular and cellular events takes place after an ischemic insult, and clinical studies have supposed that this acute response affects not only clinical outcomes but also the extent of brain injury [27]. The hypoxic ischemia model repeats many cellular parameters of brain inflammation seen in stroke. Since the mononuclear cells of cord blood produces large amounts of IL-10 [28], a potent anti-inflammatory cytokine, HUCB cells treatment may influence the cascade of inflammatory/immune events, thereby explaining the neurobehavioral and histo-logical benefits observed in this study. 

The mononuclear cord blood cells have been shown to express growth factors such as nerve growth factor [29]. Therefore, these molecular-based mechanisms may include not only immune processes mediated by interleukins but also the involvement of growth/trophic factors. In addition, the existence of endogenous neuroprotective factors in the HUCB cells can have a major role in ischemic brain recovery [30]. 

Although there are few studies on the use of stem cell therapy in neonatal hypoxia-ischemia models, there is a wide range of methods used [2, 31-34]. Ma *et al.* [33] studied the effect of transplantation of embryonic stem cell-derived cells on a hypoxia-ischemia mouse model. Their results showed that the transplanted cells signiﬁcantly ameliorated the learning and memory deﬁcits eight months post transplantation. In Ma's study [33], mice embryonic stem cells were injected directly into the lesion site. However, in our study, the HUCB stem cells were injected to rats. Intravenous administeration has the advantage of being less invasive than intracerebrally injection. Yasuhara *et al.* [32] showed that both intracerebral and i.v. transplantation of multipotent adult progenitor cells results in behavioral improvement and reduction in ischemic cell loss in hypoxic-ischemic brain injury in the neonatal rat hypoxia model. Furthermore, Pimentel-Coelho *et al.* [2] claimed that HUCB transplantation (i.p.) ameliorates animal’s performance in two developmental sensorimotor reflexes three hours after the hypoxia-ischemia insult. Thus, the route and the date of delivery are both considerable. 

In the present study, the possible therapeutic role of HUCB stem cells in a model of neonatal hypoxia-ischemia brain injury was investigated. Moreover, transplanted cells could survive and migrate into the rodent brain without immunosuppression, and ischemic rats showed improved neurological function after transplantation. The histological studies also confirmed that the labeled cells were present at the site of injury, and more HUCB stem cells were found in the cortex of hypoxia-ischemia rats than in control rats, suggesting that ischemia-induced chemotactic factors facilitate migration of HUCB stem cells. In addition, morphologic analyses showed that i.v. injection of HUCB cells signiﬁcantly reduced the severity of the infracted area in neonate brains.

Two established motor tasks, namely modified limb-placing test and corner turn test, were employed in this study. For the modified limb-placing test, the control animal's point was 16, while the scores of both the test and sham groups had significantly difference in comparison with the control group in the first and seventh days after the cell injection. However, no significant difference was observed in the test animals and the untreated control group on day 14 after injection. For second test, the rotation of the control normal animals to the right and left was 50% on the first day after injection, while the test and sham groups tended to turn to the right near 100% on the first day after injection. The comparison of the percents of the two groups and the control group indicated significant difference. No difference was observed between the test and control groups on day 14 after injection, and the percents of the rotation in the test animals were almost 50%. 

In summary, these findings attribute recovery from ischemic injury in hypoxia-ischemia neonatal rats to severe brain damage to the placement of HUCB cells in the injured area. In addition, the loss of damaged tissue in the hemisphere of the test group confirmed that cord blood stem cells can be used for treatment of hypoxia ischemic stroke. Of course, investigation of dose, timing, route of HUCB cell delivery, immunosuppression, and the use of associated therapies is necessary for clinically applied of cellular therapy, and it is considerable for optimizing neuroprotection and consequently neurobehavioral outcomes. 
